# Numerical Failure Analysis and Fatigue Life Prediction of Shield Machine Cutterhead

**DOI:** 10.3390/ma14174822

**Published:** 2021-08-25

**Authors:** Jie Li, Zengqiang Zhang, Chuang Liu, Kang Su, Jingbo Guo

**Affiliations:** School of Mechanical Engineering, Shijiazhuang Tiedao University, Shijiazhuang 050043, China; zhangzq1127@stdu.edu.cn (Z.Z.); l_c19@126.com (C.L.); sukang@stdu.edu.cn (K.S.); guojingbo66@163.com (J.G.)

**Keywords:** cutterhead, failure analysis, life prediction, crack propagation, stress intensity factor

## Abstract

This paper presents numerical failure analysis on cracking of shield machine cutterhead structure during a metro-tunnel construction. The stress intensity factors (SIFs) of surface cracks with different shapes and location angles were analyzed by a finite element simulation method based on linear elastic fracture mechanics (LEFM) theory. The ratios of variation in stress intensity factors of cracks with different shapes were analyzed. The maximum allowable crack depth of the cutterhead panel is 50.23 mm by dynamic stress calculation, and the damage tolerance criterion of the cutterhead panel was proposed. The influence of the Paris model parameter values was analyzed based on mathematical methods. It is proven that the location of the cutterhead cracking angle is mainly determined by the mixed-mode SIF. In practice, the crack section basically expanded into the semi-elliptical shape. The cutterhead structure may directly enter the stage of crack propagation due to welding defects during tunneling. The research results provide a theoretical basis and important reference for crack detection in the key parts of the cutterhead, as well as maintenance cycle determination and life prediction of the cutterhead mileage, both of which have important engineering value.

## 1. Introduction

**Figure 1:**
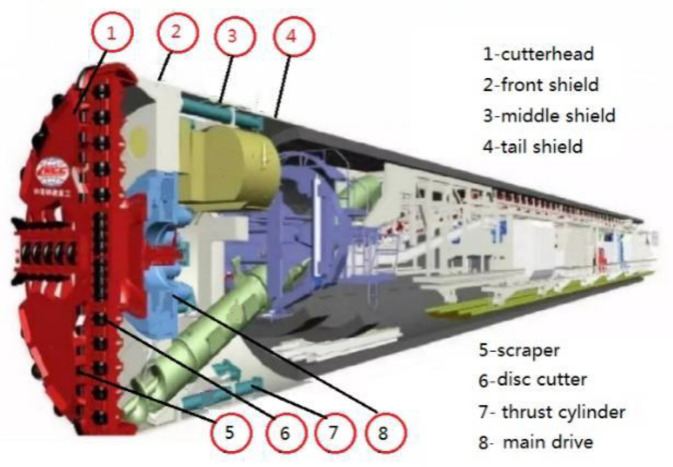
Structure of shield machine.

The cutterhead of a shield machine is generally a large welded structure, which is welded using steel plate to form a whole structure, and the corresponding position is reserved to install the disc cutters and scrapers. The structure of a shield machine is shown in Figure 1.

**Figure 2:**
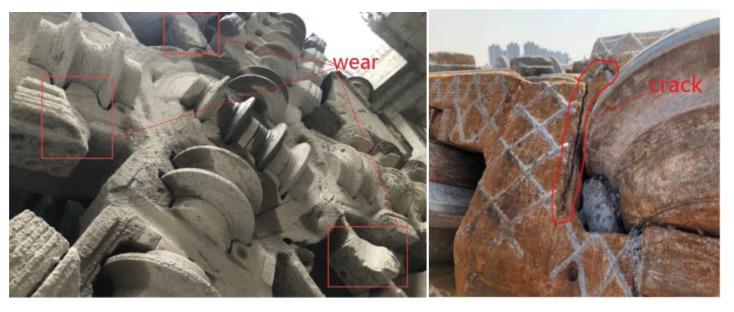
Failed cutterhead of a shield machine.

The working principle of a cutterhead is as follows: the cutterhead breaks and cuts the soil using the cutters installed on the panel with the comprehensive action of thrust and torque; the tunnel section is formed once. Generally, the cutterhead of a shield machine is welded by carbon-dioxide gas shielded welding, and an ultrasonic testing method is used for flaw detection, with the highest accuracy being Φ2 mm. Due to the extremely complex geological conditions in the process of cutterhead excavation, cracks will be initiated and propagated in small defects or weak links by the action of alternating loads, which can result in structural failure. The wear and fatigue cracks are the main forms of cutterhead failure, as shown in Figure 2. 

The cutterhead is the core component of a shield machine. Its service performance directly affects the excavation efficiency of a shield machine and the safety of the construction. Considering the complex structure of a shield machine cutterhead, the problem of reliability becomes particularly important. The static design method is predominantly used in the cutterhead structure design, and a large safety factor is involved to ensure the safety of the cutterhead structure. In the construction of hard strata, such as bedrock or solitary stone, the problems of excessive wear and fatigue cracking of the cutterhead appear easily, endangering the safety of tunneling construction.

In the fatigue life prediction of large structures such as cutterhead, Ling [1] proposed a large-scale structure life prediction method based on system dynamics, linear elastic fracture mechanics and fatigue damage accumulation theory. The predicted cutterhead driving mileage is able to meet the design objectives. Sun Wei et al. [2] studied the cutterhead panel material Q345 multi-crack propagation damage law and gave the change rule, and propagation path of stress intensity factors during the propagation process of collinear crack, parallel crack and non-parallel crack. The results lay a foundation for predicting the multi-crack propagation life of a cutterhead. Ling & Sun et al. [1,3] established the probability density function of the load distribution of a cutterhead using statistical method, and then calculated it using a rain flow counting method, and compiled the eight-level load spectrum. In the fatigue crack-growth calculation model, Liu et al. [4] put forward the small-time scale prediction model based on continuous crack-growth, and obtained better prediction results for aluminum alloy and other materials. Huo et al. [5] predicted the influence of thick plate on crack-growth behavior, based on the improved small-time scale model, and obtained the expression of constraint factor function. Due to the complex structure of a cutterhead and changeable geological environments, the cracks generated by a cutterhead are mostly composite cracks [1,3,6]. The stress intensity factors of mode Ⅰ, Ⅱ and Ⅲ of mixed-mode cracks are different, and are relatively complex crack forms.

Dicecco Sante et al. [7] studied the effect of surface corrosion on the high-cycle fatigue and low-cycle fatigue of Q345 mining wheel. When the test cycle reached 5 × 10^5^, the fatigue strength of the corroded surface decreased by 24.6% compared with the low-cycle fatigue. At the same time, the fracture behavior of the low-cycle fatigue corrosion sample and the polished sample were obviously different. Dong et al. [8] Studied the low-cycle fatigue mechanism of Q345 steel for pressure vessel, established the linear relationship between crack-tip opening displacement and crack-growth rate, and analyzed the influence of the plastic zone. Zong et al. [9] studied the fatigue crack-growth rate of bridge steel Q345qD. Based on probability statistics, the mean value and variance estimation model of parameter C and m of the Paris formula for crack-growth and the design parameters were given. The results can provide reference for the fatigue resistance design of steel structures. The Paris model was put forward by Paris and Erdogan [10] in 1963, and was further developed and improved later by many scholars. The landmark theories and formulas are Austen growth model [11], Forman model [12] and Neuman [13] crack-tip opening displacement model to calculate the crack-growth rate. Some scholars have developed the Paris model and applied it to aluminum alloy [14] and thick plate [5] and welding joint [15]. These studies enrich the application range of the Paris formula and provide a more accurate model to predict the structural fatigue life.

Some scholars [8,9] obtain material crack-growth parameters C and m by means of experiments and data statistics method. These data take material homogeneity, experimental repeatability and statistical reliability into account, and can be used as basic data for structural design and evaluation. Generally, the parameter m has a greater influence on the fatigue crack-growth life [3]. According to observations made in experiments conducted by researchers, the results of the average value analysis of general statistics are more general and representative.

Some scholars have studied the shear propagation of cracks in materials. Feng Yu et al. [16] presents an experimental study on diagonal crack width estimation of Shear-Strengthened Pre-damaged Beams with CFRP strips (SSPBCs). Several parameters including pre-damaged degree, shear-span ratio and CFRP strips spacing are considered. The crack formation of shear-strengthened undamaged or low pre-damaged beams with CFRP strips is caused by reaching the ultimate tensile strain of concrete, while that of shear-strengthened high pre-damaged beams with CFRP strips is due to the relative slip between stirrups and the concrete. The development rate of diagonal crack increases as the shear-span ratio, CFRP strips spacing, or pre-damaged degree increases. Yuya Tanaka et al. [17] investigated the shear-mode crack-growth for the fatigue strength of Ni-base superalloy. Three different types of fatigue tests were performed: (i) push-pull; (ii) pure-torsion; (iii) torsion with superposed static tension. All tests revealed non-propagation of small, shear-mode cracks.

In summary, some progress has been made in the research on large structure fatigue life prediction, and the crack fatigue life under service conditions has been predicted. The general and universal calculation flow of different geological and cutterhead forms are still lacking. Under different structures of cutterhead and different geological conditions, it is necessary to analyze the failure of the cutterhead and conduct the life prediction of the cutterhead.

**Figure 3:**
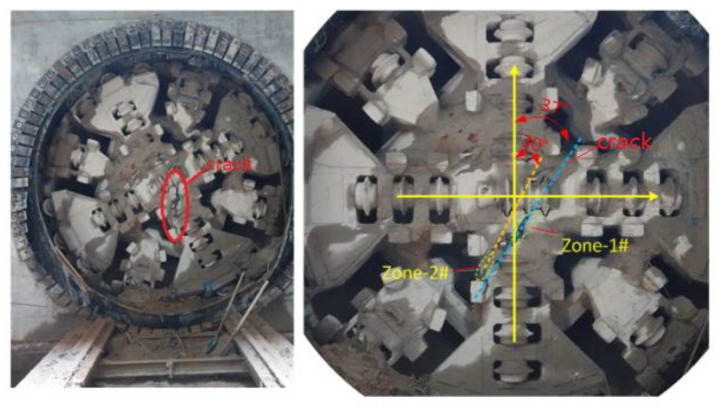
Diagram of cutterhead cracking.

A shield machine was used in the construction of a subway tunnel in Xuzhou of China. The cross-section of the tunnel is a composite stratum, and there are bad strata such as bedrock intrusion; the maximum value of uniaxial compressive strength is 122 MPa. When the tunnel section is perforated, cracking is found in the front panel, as shown in Figure 3. The crack length is about 1.25 m, and the crack depth is about 16 mm. One side is located at the welding site of the cutterhead beam and scraper (zone 2#), and the other side is located at the center cutter saddle (zone 1#). The crack is basically a straight line. The angle with the transversal direction is approximately about 53~60°.

To solve this problem, numerical failure analysis was carried out to identify the root causes, and the crack-growth law was applied to predict the crack-growth life of the cutterhead. This paper demonstrates an engineering case of structural failure due to fatigue crack on a certain type of cutterhead, which ensures the safety of the construction process and provides reference for similar structures.

## 2. Modeling and Static Analysis of Shield Cutterhead

### 2.1. 3D Modeling of Cutterhead

**Figure 4:**
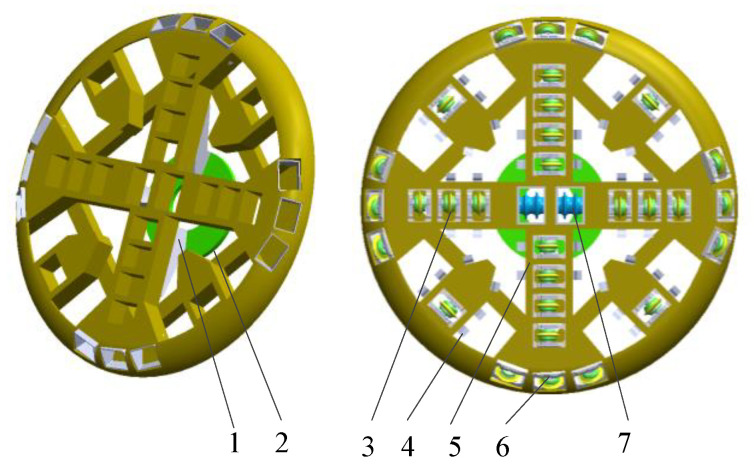
Model of shield cutterhead. 1-support; 2-flange; 3-normal disc cutter; 4-Scraper; 5-cutter beam; 6-gage disc cutter; 7-central disc cutter.

The 3D model of shield cutterhead is established in SolidWorks according to the ratio of 1:1. Without affecting the overall structural accuracy, the model of cutterhead is simplified appropriately. The characteristics of bolt hole, chamfer and rounded corner are omitted, and the weld seam is rigidized. The cutterhead model is imported into ANSYS for analysis. The overall structure of the cutterhead is welded into a whole, as shown in Figure 4. The outer side of the cutterhead is welded with scrapers. The opening rate of the cutterhead is about 30%. The rear side of the cutterhead is connected with four supporting ribs to form a whole. The bracket is connected with the back flange. The detailed parameters of the cutterhead are shown in Table 1. 

### 2.2. Static Analysis of Cutterhead

Through static analysis of the cutterhead structure, the distribution of stress-strain of the cutterhead structure is obtained. The position of the maximum stress is sought, which is set as a dangerous point. Static analysis lays a foundation for further fracture mechanics analysis.

**Figure 5:**
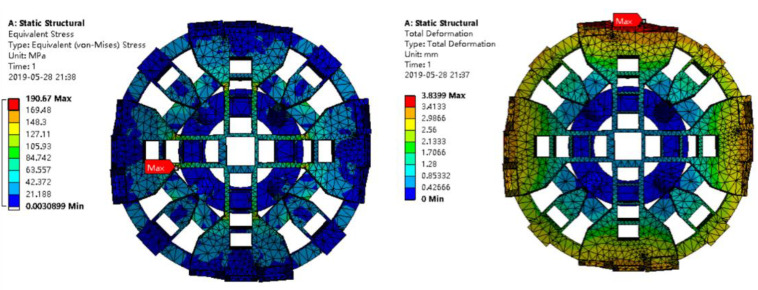
Static analysis results of cutterhead.

The whole cutterhead is imported into ANSYS Workbench and meshed by Tetrahedrons element. The mesh size is adaptive and the accuracy is medium. The number of elements is 27,742, and the number of nodes is 54,060. The average element size is 60 mm. The cutterhead boundary conditions are set as follows: the flange is added with fixed constraints, and the surface force is 0.80 MPa and the torque is 2000 N·m. The calculated results are shown in Figure 5. The figure shows that the maximum deformation of the cutterhead is 3.840 mm and the maximum stress of the cutterhead is 190.67 MPa. The cutterhead safety factor s_1_ is 1.81, which indicates the structural static safety. In Figure 5, it can be seen that the local maximum stress is 190.67 MPa, which is located on the inner side of the cutterhead beam support plate. The cutterhead structure can be simplified to beam structure, and it is easy to produce stress concentration, which is also the focus of cutterhead maintenance.

## 3. Crack Modeling and Analysis

According to the linear elastic fracture mechanics theory, a semi-elliptical surface crack is inserted at the initial cracking location. The numerical method [1] is used to solve the stress intensity factor, and the crack propagation variation law is analyzed according to the variation law of the stress intensity factor.

### 3.1. Analysis of Cracking Direction

The crack began on zone 2# of the cutterhead of the shield machine during tunneling. After initiation, the crack entered the stage of propagation and expanded continuously along the directions of length and depth.

**Figure 6:**
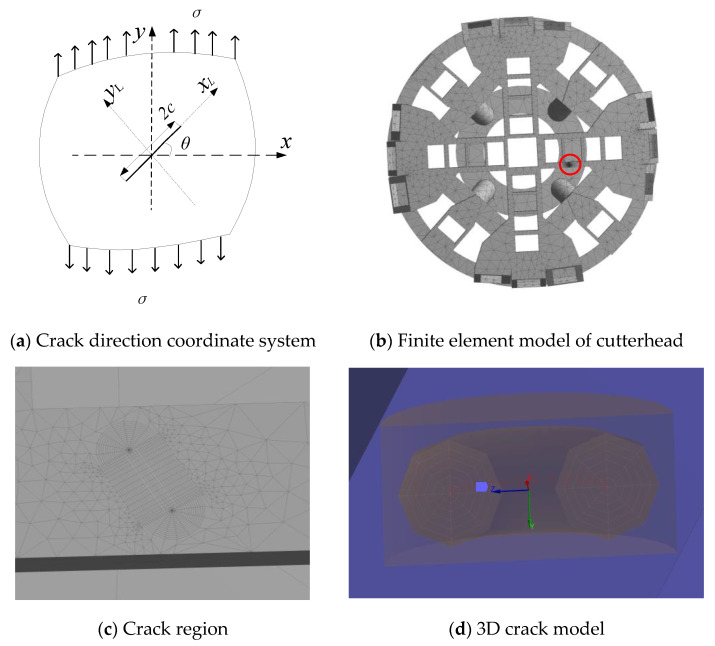
Definition of crack position angle: (**a**) Crack direction coordinate system (**b**) Finite element model of the cutterhead (**c**) Crack region (**d**) 3D crack model.

A schematic diagram of the crack position angle is illustrated in Figure 6. *xoy* is the absolute coordinate system, *x*_L_ is the direction of the crack length, *θ* is the angle between the crack length and the *x*-axis, and the crack length is 2*c*. The finite element model of the cutterhead with the same crack size (2*c* = 60 mm, *a* = 15 mm) was taken as the research object under the maximum loading condition. Considering the randomness of crack position angles, the variation rules of crack stress intensity factor at different crack position angles were analyzed. The variation law of the stress intensity factors of the cracks was obtained. The stress intensity factors of the three cracks in the range of 0~90° were calculated by taking 15° as interval.

**Figure 7:**
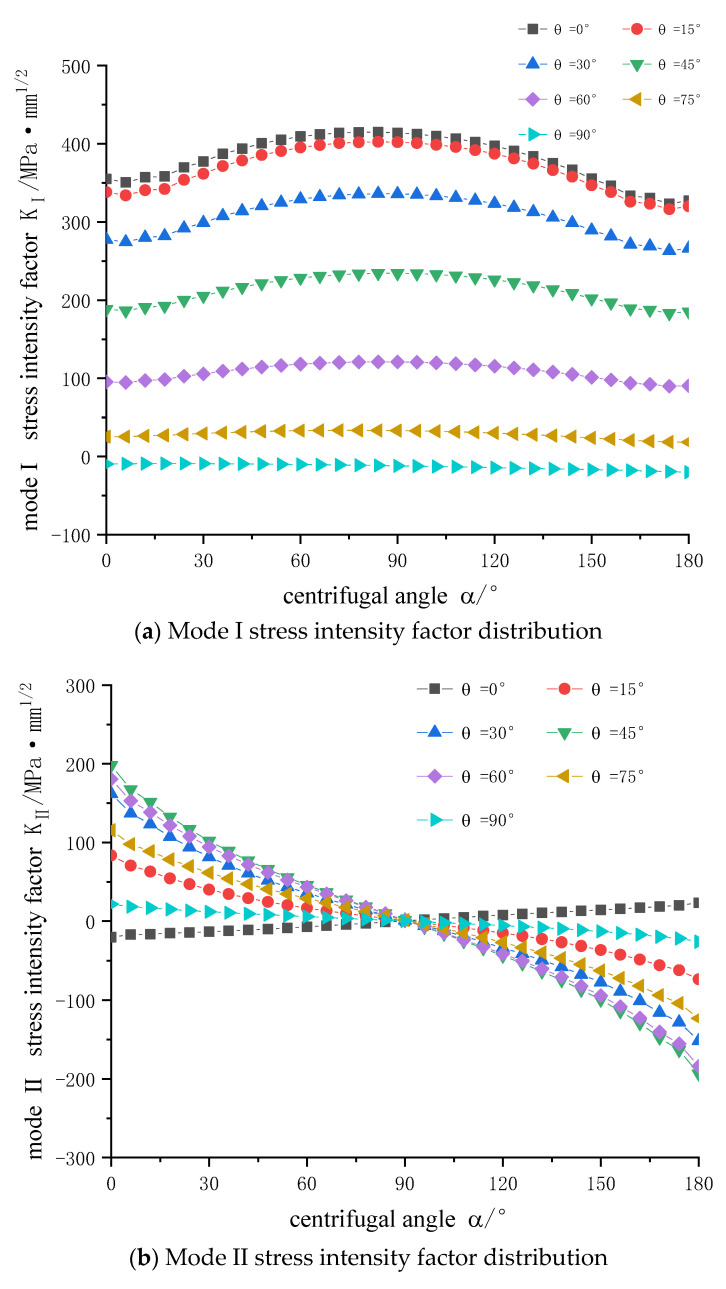

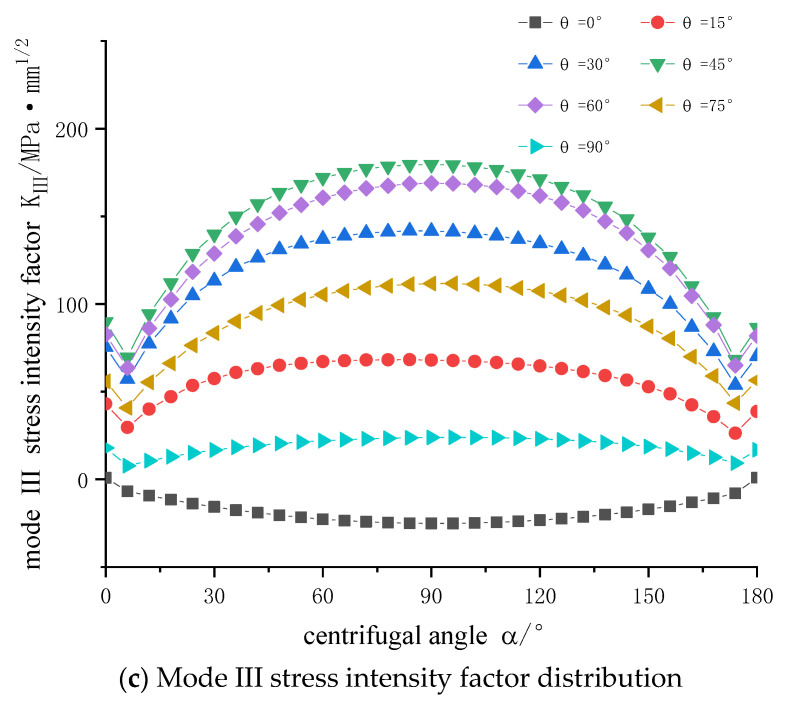
Three modes stress intensity factor distributions: (**a**) Mode I stress intensity factor distribution (**b**) Mode II stress intensity factor distribution (**c**) Mode III stress intensity factor distribution.

As shown in Figure 7, crack stress intensity factors of different crack centrifugal angles under different crack position angles are presented. 

Figure 7 demonstrates the following conclusions:

(1) The stress intensity factors of mode I are basically distributed symmetrically. With the increase of *θ* angle, the value of stress intensity factors decreases, and tends to be flat near 90°. When *θ* = 0°, the value of stress intensity factor reaches the maximum 414.87 MPa·mm^1/2^. When *θ* = 90°, the value of stress intensity factor is negative. The stress intensity factors of mode I only exists when it is open. When K_I_ < 0, it has no significance.
(2) The stress intensity factors of mode Ⅱ are basically central-symmetric with *θ* = 90°, the value decreases from left to right, and the overall value increases first and then decreases with *θ* angle. When *θ* = 45°, the maximum value is 197.88 MPa·mm^1/2^.
(3) The stress intensity factors of mode Ⅲ are basically symmetrical. The absolute values of the stress intensity factors increase first, and then decrease, with the increase of *θ* angle. Except for *θ* = 0° all the stress intensity factors are positive, indicating that the increase of *θ* angle changes the tearing direction, and the maximum value is 179.49 MPa·mm^1/2^ at *θ* = 45°.

**Figure 8:**
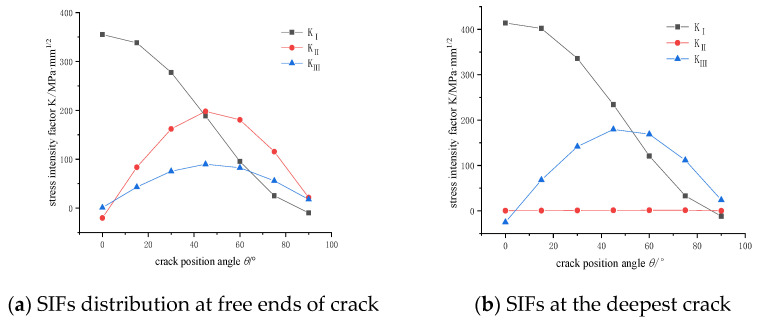
Stress intensity factor distribution of cracks: (**a**) SIFs distribution at free ends of crack (**b**) SIFs at the deepest crack.

The main driving force of the crack propagation direction comes from the stress intensity factor at the free ends of the crack surface [18]. As shown in Figure 8, the stress intensity factors of mode I and Ⅱ are always more than two times of mode Ⅲ in the range from 0~75°. Angle ranges from 75~90° are about twice as much. From the comprehensive analysis, it can be seen that the main form of crack cracking is the mixed mode. 

The formula of maximum circumferential stress [19] in the polar coordinate system is established as

(1) $$\left\{ \begin{array}{l} \sigma_{r}=\frac{1}{2\sqrt{2\pi r}}\left[K_{I}\left(3-\cos\theta \right)\cos\frac{\theta }{2}+K_{II}\left(3\cos\theta -1\right)\sin\frac{\theta }{2}\right]\\ \sigma_{\theta }=\frac{1}{2\sqrt{2\pi r}}\cos\frac{\theta }{2}\left[K_{I}\cos^{2}\frac{\theta }{2}-\frac{3}{2}K_{II}\sin\theta \right]\\ \tau_{r\theta }=\frac{1}{2\sqrt{2\pi r}}\cos\frac{\theta }{2}\left[K_{I}\sin\theta +K_{II}\left(3\cos\theta -1\right)\right] \end{array}\right.$$

where, cos(θ/2)≠0 that is θ≠±π, and two free surfaces are not considered. The radius *r* does not tend to zero, otherwise infinity will occur, which means the crack tip will not be considered. The condition for circumferential stress to extremum is

(2) $$\frac{\partial \sigma_{\theta }}{\partial \theta }=0$$

The simplified formula is

*K*_Ⅰ_ sin*θ*_0_ + *K*_Ⅱ_(3cos*θ*_0_ − 1) = 0 (3)

When *K*_Ⅰ_ and *K*_Ⅱ_ values are introduced, the solution of *θ*_0_ can be obtained. From the actual crack direction, the crack position angle is about 53~60°. When *θ* = 53° is brought, *K*_Ⅰ_ = 138.66 MPa·mm^1/2^ and *K*_II_ = 192.61 MPa·mm^1/2^, we obtained *θ*_0_ = 57.58°. The relative error of the solution is 8.6%. When *θ* = 60°is brought, *K*_Ⅰ_ = 95.455 MPa·mm^1/2^ and *K*_II_ = 180.55 MPa·mm^1/2^, and *θ*_0_ = 60.84°is obtained, the relative error is 1.4%. The main factors causing errors are the influence of mode Ⅲ stress intensity factor *K*_Ⅲ_. Moreover, the closer the numerical value of the stress intensity factor is to the real crack-position angle, the smaller is the error.

### 3.2. Crack Propagation Law with Different Shape Ratio

The actual crack shape changes during the crack-growth process [20], which is due to the inconsistency of crack-growth rate in the depth and length directions. It is necessary to reveal the crack propagation behavior and analyze the distribution of crack stress intensity factors of cracks with different shapes.

**Figure 9:**
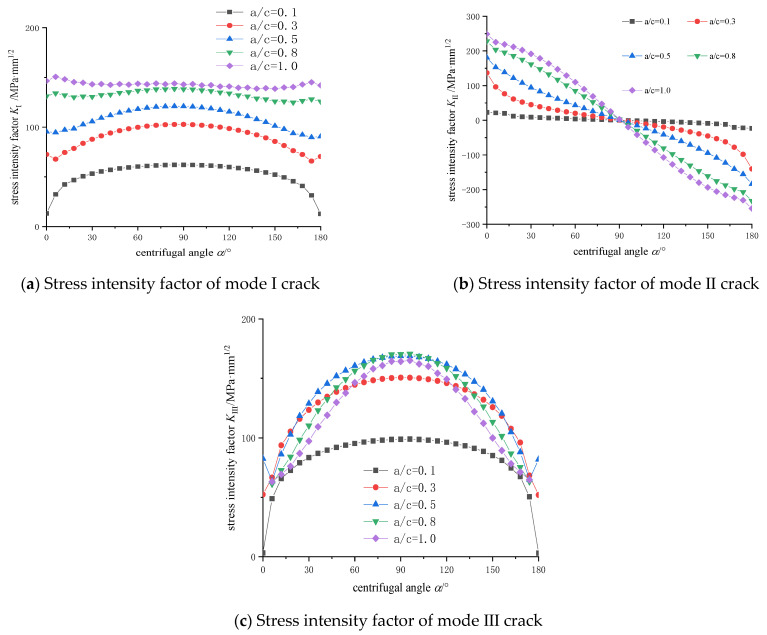
Stress intensity factor distribution of cracks with different shape ratio: (**a**) Stress intensity factor of mode I crack (**b**) Stress intensity factor of mode Ⅱ crack (**c**) Stress intensity factor of mode Ⅲ crack.

First, a cutterhead model with a semi-elliptical crack is established at the zone 2#. The crack length axis c = 30 mm is taken as the same, the crack depth a is constantly changing. The shape ratio *a/c* are 0.1, 0.3, 0.5, 0.8 and 1.0, then the simulation model of the position angle 60° crack is sequentially established. The distribution of stress intensity factors at different crack shape ratios is analyzed and the results are shown in Figure 9.

From the stress intensity factors curves in Figure 9, insights are gained as follows:

(1) The stress intensity factors of mode I crack are basically symmetrical, and range from 50 to 150 MPa·mm1/2. With the increase of the crack shape ratio, the values of stress intensity factors increase gradually. When the crack is very shallow, the main propagation is depth growth, the length growth is secondary; when the crack approaches circular (a/c = 1.0), the stress intensity factor of crack is basically linear.
(2) The stress intensity factors of mode Ⅱ crack are basically 90° center symmetry, indicating that the direction of crack slip has changed. As the shape ratio increases, the values of the stress intensity factor increase gradually. It shows that the closer to the circle, the faster the expansion speed.
(3) The stress intensity factors of mode III crack are symmetrical, increase firstly and then decrease. Except for the narrow crack of a/c = 0.1, the other crack stress intensity factors are close to each other, and the maximum value is near 150 MPa·mm1/2. When a/c = 0.8 and 1.0, the free ends of the crack appear singular, and the endpoint singular values are discarded. The crack-growth path is generally controlled and affected by many factors, which is one of the key research directions in the next stage. This paper offers some preliminary discussions. For example, at the zone #1, the cracks are mainly mode I cracks, so the overall crack-growth trend is linear, but the local path twists and turns under the control of mode II and mode III stress intensity factors, due to the structure and load. However, according to the law of crack propagation path, the corresponding crack arrest structure design can be carried out to prolong the structural life.

## 4. Crack Propagation Life of Cutterhead

### 4.1. Initial Crack Size Determination

The material of a cutterhead is mainly Q345D steel, and the cutterhead is assembled as a whole structure through welding. The physical parameters of cutterhead material Q345D are shown in Table 2, where D indicates that the V-notch impact test energy of the material is greater than 27 J at −20 °C. Problems such as long weld length, plate thickness and difficult penetration may lead to welding defects. The sensitivity of the ultrasonic test (UT) instrument in engineering application is mostly Ф2 mm [3], which can detect the initial crack size of 2 mm or more. After a long period of heavy load, vibration and other comprehensive effects, the welding defects gradually expand until the strength of the cutterhead is insufficient and the fracture/failure occurs.

Initial surface crack size refers to the crack size that begins to calculate the life of the crack propagation stage, and can be evaluated by non-destructive testing. In applying engineering considerations, the crack size should be determined comprehensively on the basis of considering the allowable defect degree of structure, the accuracy of existing instruments and the technical level of operators. The initial crack depth used in engineering *a*_0_ is 0.5 mm [4]. According to the conventional ultrasonic testing method, the crack length 2*c*_0_ is 2 mm.

### 4.2. Criterion of Crack Damage Depth Tolerance

The critical crack size is the allowable maximum crack size of a cutterhead structure, which is generally expressed by *a_c_*. The critical crack size is determined by *K* criterion, and thereby can be obtained as follows:

(4) $$a_{c}=\frac{1}{\pi }{\left(\frac{K_{\mathrm{IC}}}{\alpha n\sigma_{\max}}\right)}^{2}$$

**Figure 10:**
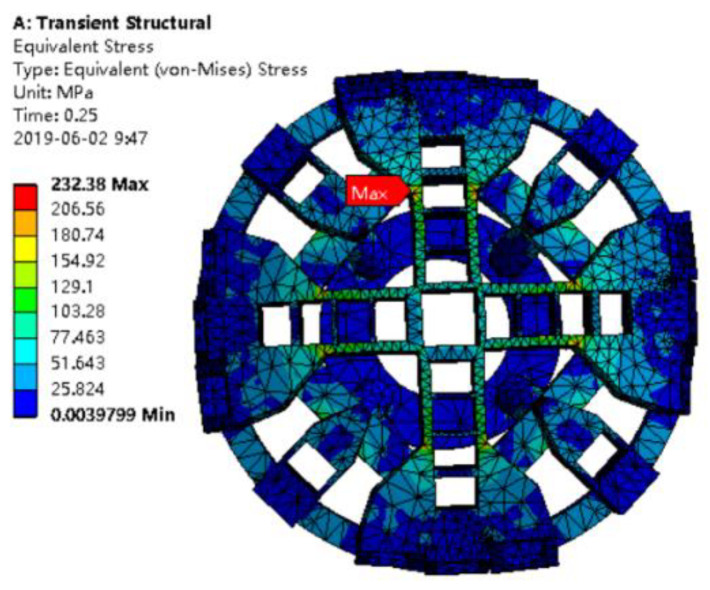
Transient dynamics nephogram.

In Formula (4), the crack shape coefficient *α* is 1.1. In reference [20], the safety factor s_1_ is 2 and the Q345 fracture toughness value *K*_IC_ is 203.08 MPa·m^1/2^. As seen in Figure 10, the maximum stress σ_max_ is 232.38 MPa. When introducing the above values into Equation (4), the damage tolerance value of the cutterhead crack depth direction can be obtained as follows:

$$a_{c}=\frac{1}{\pi }{\left(\frac{203.08}{1.1\times 2\times 232.38}\right)}^{2}\times 1000=50.23\mathrm{mm}$$

The criterion of depth damage tolerance of the cutterhead panel is proposed, which provides a basis for further calculation of crack-growth life.

### 4.3. Analysis of Fatigue Crack-Growth Rate Parameters

In 1960′s, the Paris model [10] was established to calculate the crack-growth life. The results of previous research, references [3,10], show that the fatigue parameters *C* and *m* are dispersive. The fatigue parameters *C* range from 1.0619 × 10^−13^ to 3 × 10^−13^ and *m* range from 3.07 to 4.76.

Crack depth and length direction propagation rates are:

(5) $$\begin{array}{l} da/dN=C_{a}{\left(\Delta K_{eqa}\right)}^{m}\\ dc/dN=C_{c}{\left(\Delta K_{eqc}\right)}^{m}\\ C_{c}=0.9^{m}C_{a} \end{array}$$

**Figure 11:**
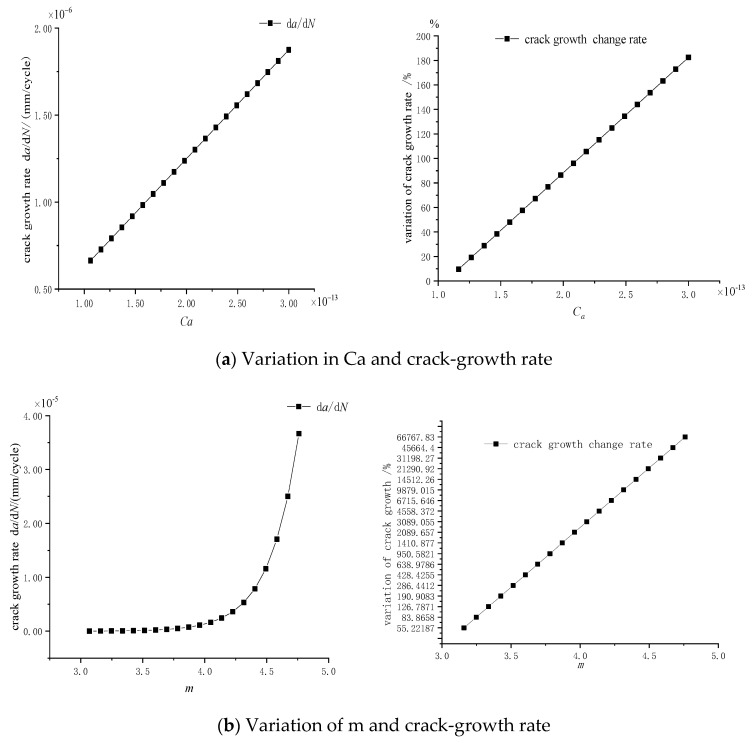
The effect of parameters changes on the growth rate: (**a**) Variation in Ca and crack-growth rate (**b**) Variation in m and crack-growth rate.

The crack propagates in both depth and length directions. According to Formula (5), the partial derivatives of C*_a_* and m are calculated, respectively, and the effect of the partial derivatives on the crack-growth rate is investigated. As shown in Figure 11, taking *m* = 4.0 and Δ*K*_eq*a*_ = 50 MPa·mm^1/2^, and the rate increases linearly with C*_a_* and exponentially with *m*. When *m* less than 4.0, the rate changes relatively gently. The crack-growth rate increases rapidly with *m* increasing when m is bigger than 4.0. When the stress intensity factors of the crack ranges are in the same interval, the depth growth rate is larger than the length expansion rate. It is recommended that *C* should be less than 2 × 10^−13^ and *m* should be less than 4.0, which tends to be safe.

### 4.4. Prediction Model of Crack Propagation Life

**Figure 12:**
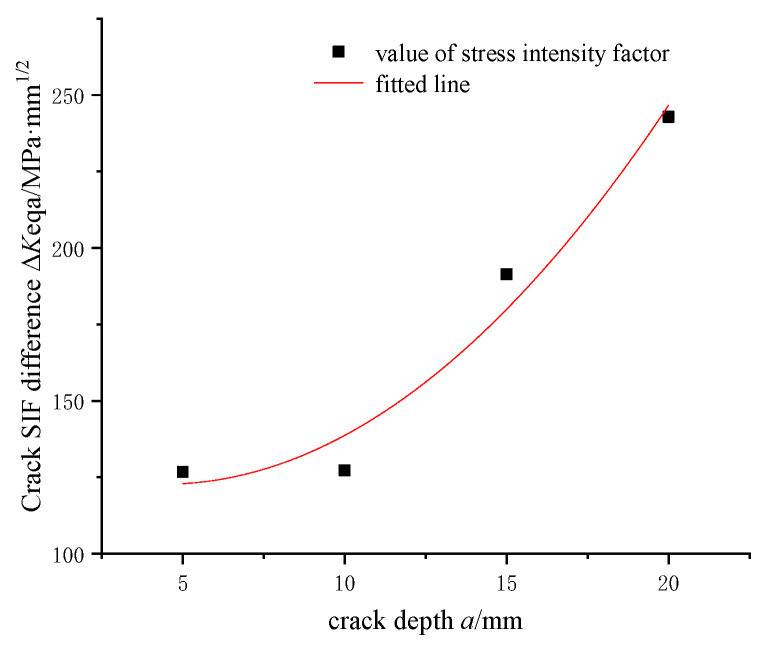
Fitting curve of crack stress intensity factor with crack depth.

The cutterhead welding defects have experienced the process of crack initiation and expansion during the excavation process. Crack initiation life accounts for the vast majority of fatigue life, even more than 80%. Based on the conditions of the engineering site application, the cutterhead crack is generated during the tunneling process. The deepest crack stress intensity factor of the difference depth *a* between Δ*K*_eq*a*_ drive crack is extended into depths. The cracks always keep shape with semi-ellipse [5]. The shape ratio *a*_0_/*c*_0_ = 0.5 was determined according to the initial crack. The stress intensity factor values of the crack at four different depths are calculated, and using the method of quadratic equation fitting, the curve is shown in Figure 12. Meanwhile the quadratic fitting formula (6) is obtained as follows, and the fitting coefficient *R*^2^ is 0.969.

Δ*K*_eq*a*_ = 135.562 − 4.483*a* + 0.509*a*^2^ (6)

According to reference [4,10], the calculation flow of cutterhead crack-growth life is presented as Formula (7) based on the Paris model.

(7) $$N=\int_{N_{0}}^{N_{c}}dN=\int_{a_{0}}^{a_{c}}(\frac{1}{da/dN})da=\int_{a_{0}}^{a_{c}}\frac{1}{C{\left(\Delta K_{eqa}\right)}^{m}}da$$

Using the values for *C* as 2 × 10^−13^ and m as 3.5, we then introduce them into Formula (7) to obtain the load cycle number *N*.

Each rotation of the disc cutter is applied as a cycle load excitation to the crack of the cutterhead. From the rotation center of the cutterhead, the crack position radius at the cutterhead *r*_1_ is 800 mm. Assuming that the disc cutter rolls purely, the disc cutter rotation speed *n*_c_ can be expressed as follows:

(8) $$n_{c}=\frac{n\times r_{1}}{d_{0}/2}=\frac{n\times 800}{216}=3.70n$$

The average rotation speed *n* and penetration *p* of the cutterhead are 5 r/min and 8 mm/cycle, respectively [1]. Then, the crack load excitation time cycle can be calculated by T = 60/(3.7 × 5) = 3.24 s. So, the total working time *t* is expressed by

*t* = *N* × T = 2.7001 × 10^6^ × 3.240 = 8.748 × 10^6^ s (9)

So far, the shield machine tunneling speed *v* is

*v* = *w* × *p* = 40 mm/min (10)

Therefore, the fatigue life of the stable crack propagation stage is converted to the tunneling mileage *L* as follows:

*L* = *v* × *t* = 8.748 × 10^6^ × 40 ÷ 60 = 5.832 km (11)

It can be concluded that this type of cutterhead will break completely when the tunneling mileage is 5832 km. In this subway tunnel project, the tunneling mileage of the metro section is about 2 km. It is assumed that the structure of the cutterhead may enter the stage of crack propagation directly due to welding defects. According to the above calculation, it can be concluded that the cutterhead will have cracks in the process of tunneling, and the crack depth of the cutterhead is about 17.054 mm. The depth of the crack is consistent with the actual situation. The appearance of fatigue crack on the cutterhead will lead to the decrease in cutterhead strength, and will eventually cause fatigue crack failure of the cutterhead. Therefore, it is necessary to strengthen the flaw detection of the cutterhead where cracks easily occur. When the conventional ultrasonic flaw detection method cannot meet the requirements, a higher precision flaw detection method should be adopted.

## 5. Conclusions

The stress intensity factors at different positions and angles of cracks were analyzed using numerical failure analysis of the shield machine cutterhead. It is concluded that the crack direction is mainly driven by mode Ⅰ-Ⅱ compound stress intensity factors at the free ends of the crack. The angle relative error between the theoretical value of 53~60° ranges from 8.6% to 1.4%. The crack shape ratio *a/c* between 0.3 and 0.8 in the crack propagates, and the crack fracture surface always keeps semi-elliptical shape. It is under the action of mode Ⅰ–Ⅱ composite stress intensity factors at the free end of the crack that the cutterhead finally produces a crack and the crack begins to expand.

The maximum allowable crack depth is calculated to be 50.23 mm through transient dynamic analysis. Criterion for crack fracture damage tolerance of a shield machine cutterhead plate is proposed. The equivalent stress intensity factor is obtained by composite criterion, and the quadratic function relationship between crack depth and stress intensity factor is fitted. This type of cutterhead will break completely when the tunneling mileage is 5832 km.

This structure of the cutterhead may have welding defects, and there is no crack initiation stage basically, and it directly enters into the crack-growth stage. The actual crack depth of the cutterhead is about 16 mm, while the calculated result is 17.054 mm. It is basically consistent with the result of the calculation, which proves the correctness of the method. It is suggested to strengthen the detection of cracks or welding defects in the dangerous position where the cutterhead is prone to crack, so as to effectively prevent the cutterhead from cracking and failure.

## Figures and Tables

**Table 1:** Cutterhead profile.

Main Technical Parameters of Cutterhead	
Excavation diameter/mm	Φ 6280
Cutterhead material	Q 345 D
Total weight/t	About 75
Number of 17-inch single-edged cutters diameter *d*_0_/mm	30/Φ 432
Number of 19-inch double-edged cutters diameter *d*_1_/mm	2/Φ 483
Scraper	40

**Table 2:** Physical parameters of Q345D material.

Serial Number	Performance Index	Numerical Value
1	Density	7850 kg/m^3^
2	Elastic modulus	210 GPa
3	Poisson’s ratio	0.3
4	Yield strength *f_y_*	345 MPa
5	Ultimate tensile strength *f_u_*	500 MPa
6	Breaking threshold Δ*K*_th_	201.12 MPa·mm^1/2^
7	Fracture toughness *K*_ⅠC_	6270.8 MPa·mm^1/2^
8	Thermal conductivity	48 W/m·K
9	Coefficient of linear expansion	1.2 × 10^−5^
10	Mass heat capacity	480 J·m^−1^·K^−1^

## Data Availability

Data sharing not applicable.
